# *Individual speckle diffraction* based 1D and 2D Random Grating Fabrication for detector and solar energy harvesting applications

**DOI:** 10.1038/srep20501

**Published:** 2016-02-04

**Authors:** Jayachandra Bingi, Vadakke Matham Murukeshan

**Affiliations:** 1 Center for Optical and Laser Engineering (COLE), School of Mechanical and Aerospace Engineering, Nanyang technological University, SINGAPORE 639798

## Abstract

Laser speckles and speckle patterns, which are formed by the random interference of scattered waves from optically rough surfaces, have found tremendous applications in a wide range of metrological and biomedical fields. Here, we demonstrate a novel edge diffraction phenomenon of individual speckle for the fabrication of 1D and 2D micron and sub-micron size random gratings. These random gratings exhibit broadband response with interesting diffusive diffraction patterns. As an immediate application for solar energy harvesting, significant reduction in transmission and enhanced absorption in thin “Si-random grating-Si” sandwich structure is demonstrated. This work has multifaceted significance where we exploited the individual speckle diffraction properties for the first time. Besides the solar harvesting applications, random gratings are suitable structures for fabrication of theoretically proposed random quantum well IR detectors and hence expected that this work will augur well for such studies in the near future.

Speckles or speckle pattern are formed when a coherent beam is incident or transmit through optically diffusive (rough) surfaces or in a random medium^1^. The speckle size and height are controllable using suitable optics where as its shape is completely arbitrary. Depending on their mode of generation and detection, speckle patterns are categorized into subjective, objective and near field speckles. Speckle pattern is chaotic and contain the very important information about the object or surface from which it is generated^2^. On this basis, speckle patterns are exploited in different measurements such as Surface deformation, position, roughness, displacement, the shape of objects (Imaging), vibration and flow measurements in metrology, medicine, astronomy and defense applications^3^,^4^,^5^,^6^,^7^,^8^,^9^. Most of these techniques are based on the particulate nature of speckle (Size, intensity and distribution) and the collective wave phenomena of speckle patterns (Speckle pattern interference and diffraction)^10^,^11^. As far as the single speckle is concerned only the size and intensity are the main parameters from the application point of view. The wave properties of an individual speckle are not much focused, which can pave way for new type of measurement techniques and related applications. On the other hand, numerical, theoretical and few experimental works on random gratings have been reported in the literature, where the randomly placed grating in fibers resulted in the light localization^12^, broadband reflection^13^ and lasing^14^. The periodic random grating is shown to be significant for lateral displacement measurement and as encoders of cryptography^15^. More importantly, random gratings are (theoretically and by simulation) proved significant as an IR photo detector (Si random grating)^16^ and for optical coupling in solar cells^17^

In this paper, we introduce a novel individual speckle diffraction phenomenon and explore the possibility of utilizing it by generating the diffraction patterns for random grating fabrication. As an immediate application, we use these diffraction patterns to fabricate novel 1D and 2D random gratings by photolithography, where the diffraction pattern of each speckle forms a micron or sub-micron size grating. A randomly arranged micro grating ensemble is termed as random grating whereas the micro grating could be 1D or 2D. Finally, we demonstrate the induced reduction in transmission or enhancement of % extinction in a “Si-Random grating-Si” sandwich structure that can find solar cell based energy harvesting applications.

## Results and Discussion

### Fabrication of 1D and 2D random gratings by individual speckle diffraction

**Figure 1:**
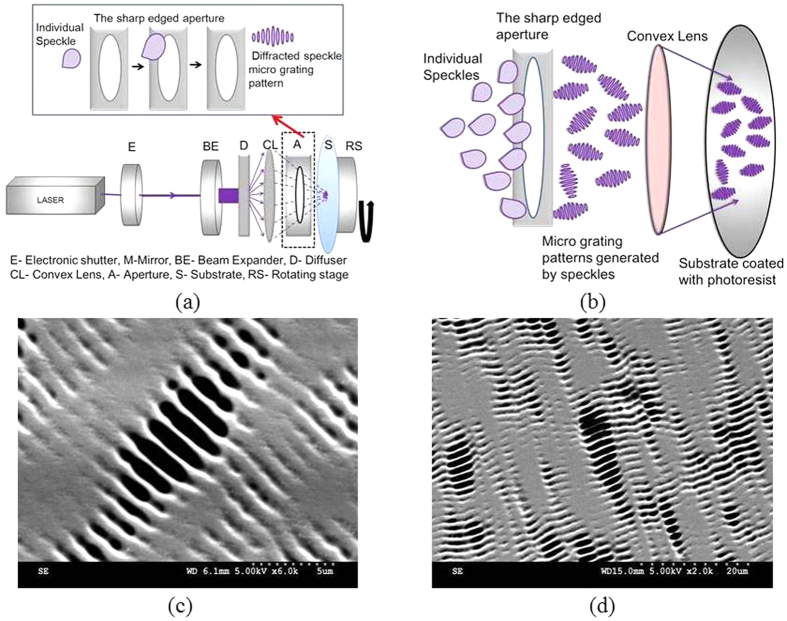
(**a**) The schematic showing the experimental se up and the inset shows the single speckle diffraction at the sharp edged aperture, (**b**) The experimental configuration in which the random micro grating ensemble (random grating) printed on the photo resist (**c**) The SEM image of micro grating generated by single speckle diffraction, (**d**) The SEM image of the random grating. (The SEM images are taken and figures are edited by Jayachandra Bingi).

The basic configuration of the experimental setup shown in Fig. 1a is based on the proposed methodology and consists of three major steps. One, generation of laser speckles with a linearly polarized laser light. Second, generating the diffraction pattern of each individual speckle by using a suitable aperture (A) with sharp edge. Third, projecting these speckles onto the substrate (S) coated with photoresist through a convex lens CL (f = 25 mm and Ø = 25 mm). Here the exposure time and the beam diameter are controlled by electronic shutter (E) and the beam expander (BE) respectively. This process is followed by the development of photoresist after the exposure. The speckles are generated by ground glass plate or diffuser (D), when the laser (linearly polarized) of 354 nm wavelength incident on it. These speckles are incident on the circular metallic aperture (A) of 4 mm diameter and 600 μm width. In this case, the formed speckle which has an average size of few microns undergo the diffraction and create a micro pattern. Inset of Fig. 1a shows the simple schematic on the diffraction pattern generation from a single speckle. Practically, the speckle pattern generated at the ground glass is a statistically distributed ensemble. As shown in Fig 1b the ensemble of speckles fall on the lens-aperture assembly, create an ensemble of the randomly distributed diffraction patterns which is projected on to the substrate coated with the photoresist (AZ7220). Hence the random grating (random distribution of micro gratings) is fabricated on the photoresist. Figure 1c is the SEM image (Scale is 5 μm) of the single speckle diffraction pattern printed on the photoresist. Figure 1d shows the SEM image of the random grating with 1D micro grating structures. The reasons behind the choice of the circular aperture and dependence of other parameters are detailed in *supplementary data section 1*

The size of the micro grating is easily tunable by varying the mean speckle size generated at the ground glass. The density of the micro gratings can be adjusted by controlling the number of speckles falling on to the lens-aperture assembly. The speckle density can be tuned by varying the D to CL distance in the experimental set up (Fig. 1a). This is based on the simple fact that if the diffuser is kept at larger distances from the plane of lens (CL), the number of speckles falling on the lens reduces. Bringing the diffuser nearer to the lens forces more speckles to enter and hence increasing the density of random microscale gratings in the pattern.

Though pitch of each grating looks similar, they are actually not the same, as detailed using the surface profile and SEM analysis. Further, the average pitch size of gratings inside the speckle is tunable by varying the width of the aperture. As the aperture’s edge sharpness affect the individual speckle diffraction, we observed the clear variation of average grating pitch from 1.45 μm to 1.08 μm when the aperture width is changed from 600 μm to 800 μm, respectively (the average is taken over 50 speckle gratings in each sample). (*The experimental data on density and pitch tunability is given in the supplementary information section 2*).

Even though the incident laser beam is a linearly polarized plane wave, it is fragmented into speckles at diffuser where each speckle pattern, with speckles having different size and shape, is formed due to the randomly interfered waves originated from the diffuser. The individual speckle is not understood completely in terms of its diffraction properties, effect of geometrical shape of apertures and shape dynamics. The fundamental understanding of speckle patterns reveals that pattern follows the Gaussian statistics with a well-defined mean speckle size. The size of an individual speckle increases with the distance which is a signature of wave divergence^18^.

**Figure 2:**
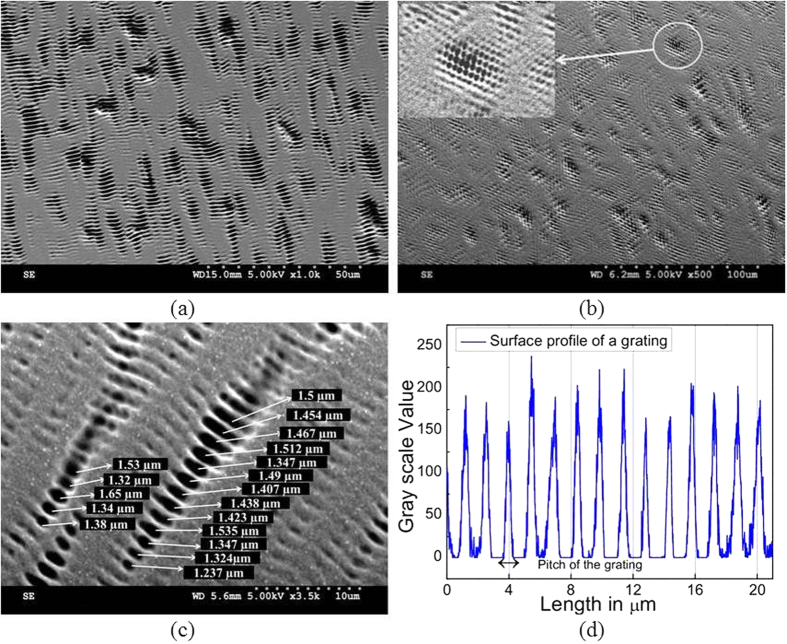
(**a**,**b**) The SEM images of the 1D and 2D random grating fabricated on photoresist. (**c**) Shows the pitch fluctuation in an individual random grating. (**d**) The surface profile of a single grating which shows the random variation of pitch (SEM images are taken by Jayachandra Bingi).

Hence, the physical problem here we deal is the edge diffraction of a single speckle. From Fig 1c, it can be observed that a spike like symmetrical energy spectrum or pattern is formed, which is perpendicular to the edge. Figure 2a shows that 1D random grating patterned on the photoresist are not of the same size because of the statistical size distribution of the speckle pattern incident on lens-aperture assembly. Figure 2b shows the 2D random gratings that are fabricated by using multiple apertures of 1.5 mm diameter in a single pupil. Here, the pupil contains 10 apertures of 1.5 mm diameter each in an area equal to 2.5 cm^2^, where the diffracted speckles from each aperture form the 2D random grating patterns.

As shown in Fig. 2c, it can be inferred that the grating pitch varies throughout both in the case a single grating or across different gratings that are patterned. This is further confirmed by Fig. 2d which represents the surface profile measured across the grating (Fig. 2c) as an example. In short, these gratings cannot be termed as ordinary gratings with constant pitch throughout. Rather, they indicate random variation of the pitch in a grating and across different gratings. In addition to these, the positional and size randomness of the random gratings (part of it can be attributed to the nature of speckles) also contribute and are quite obvious from SEM images.

### Optical properties of Random gratings

**Figure 3:**
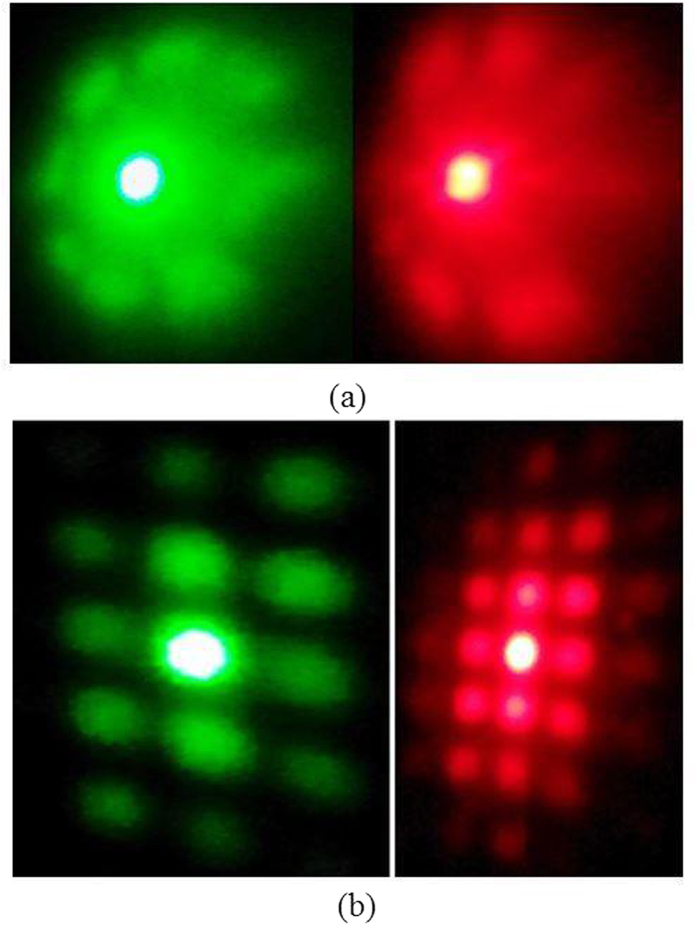
(**a**) The diffraction pattern generated by the 1D random grating at 532 nm and 632 nm, (**b**) The diffraction pattern by 2D random grating at 532 nm and 632 nm.

The optical grating is a dispersion element that splits the incident electromagnetic beam into components and forms the particular diffraction pattern. Random gratings are significant in terms of their unusual diffraction patterns. Figure 3a shows the diffraction pattern recorded when the 1 mm diameter laser beam incident on the 1D random grating (Fig. 2a) in the transmission configuration. The diffraction patterns are recorded at the 532 nm and 632 nm. The diffraction pattern has an unusual wheel like shape which could be a superposition of diffraction patterns from 1D micro gratings oriented in different directions. It is clear from the Fig 3a, besides the diffraction there is diffusion like wave spread in the case of both wavelengths (532 nm and 632 nm) which is a natural optical property of a random medium.

Figure 3b shows the diffraction pattern generated by the 2D random grating with the same experimental configuration (In transmission mode). The diffraction pattern is a 2D array of spots with some diffused noise. From Fig 2b it is clear that the micro gratings within the 2D random grating are nothing but a mesh like structure. It’s a well-known fact that these mesh like gratings generally result in such square array diffraction patterns as shown in Fig 3b. The diffusive component is less in the case of 2D random grating when compared to 1D random grating which is very clear from the well-defined array diffraction pattern.

**Figure 4:**
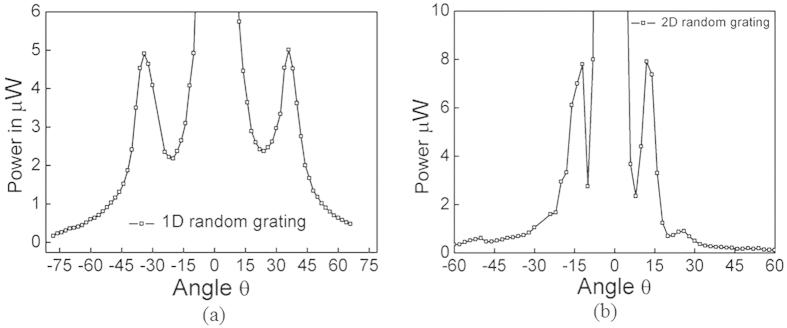
The planar angular power distribution curve of (a) 1D random grating (b) 2D random grating.

Both random gratings are exhibiting the symmetrical diffraction patterns. From the diffraction theory^19^ the average spacing in these gratings is calculated from the observed diffraction patterns. The average spacing values are 0.91 and 2.4 μm for 1D and 2D random gratings respectively. Further, the angular power distribution of the diffraction pattern has been calculated at the plane perpendicular to the gratings axis. Figure 4a,b show the power distribution spectra of 1D and 2D random gratings, respectively. The magnitude of power distribution is dominant in the 2D grating (first order peak power is 5 μW, 8 μW in 1D, 2D random gratings respectively) whereas the angular power spread in 1D random grating is 2 times that of 2D random grating. The angular positions of the first order diffracted spots are at 35° and 12° in 1D and 2D random gratings, respectively. The effective diffraction efficiency of first order with respect to 0^th^ order is calculated as ~8.67% and ~12.7% respectively for 1D and 2D random gratings. Here in the presented work, the gratings are written on a photoresist (AZ7220) whose refractive index is 1.7. The same type of gratings with high index materials^20^ can result in better efficiencies. Multi-layer^21^ and the metamaterial based random gratings can also expectedly improve the diffraction efficiencies.

**Figure 5:**
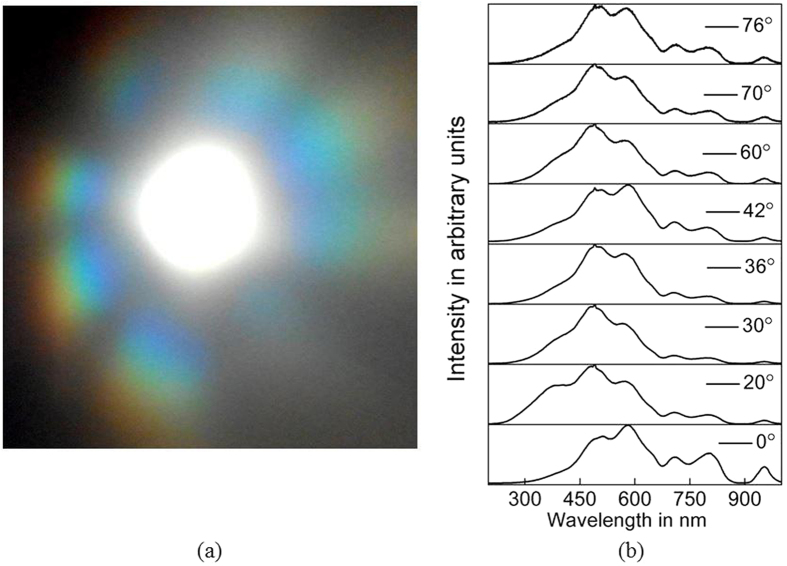
(a) Broad band light diffraction profile, (b) The spectral profiles at different angles, showing the broadband diffraction ability of 1D random gratings.

It is clear from the above observations that the pattern observed in the case of 1D random grating consist of two optical contributions, one is a diffracted light component and the other is a diffusive light component. The overlap of these optical contributions can be tested by measuring the spectral profile at different angles in the plane of perpendicular axis. Figure 5a shows the diffraction pattern created by the 1D random grating when an incoherent broadband light incident on it. It is clear from the figure that the 1D random grating creates a wavelength dispersion spectrum. Figure 5b shows the spectral profile variation at different angles. It is clear that the profile is varying in a systematic way, but still the broadband nature of the light persists. This also shows the broadband response of the 1D random grating. Besides the broadband response, the wider angle diffraction and diffusion of 1D random grating and higher optical power diffraction ability of 2D random grating are most significant in the application point of view^22^.

### The enhanced absorption in Si-Random grating - Si sandwich structure

**Figure 6:**
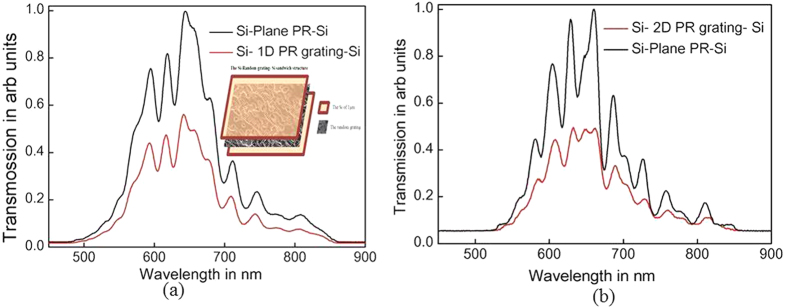
The spectra showing the reduced transmission of (a) Si-1D-Si (inset showing the Si-Random Grating –Si sandwich structure), (2) Si-2D-Si structures.

Considering the observed properties of the random gratings, it is clear that the random gratings are useful as surface patterns or the sandwiched patterns (**Si-Random grating-Si**) to create improved absorption (reduced transmission) due to scattering and absorption for solar harvesting in layered thin film structures. Here we fabricate the 1D and 2D random grating patterns of photoresist with ~500 nm thickness sandwiched between 1 μm Si (Amorphous) films. We study the transmission properties introduced by the grating pattern in the plane Si slab. The area of the pattern is 10 mm and the photoresist refractive index is ~1.7. Figure 6a,b shows the transmission spectra of broadband light of “Si-Plane PR-Si” (black curve) and the “Si- 1D and 2D PR random grating-Si” (red curve) respectively. From Fig. 6 the transmission is reduced and hence the absorption is enhanced due to the grating pattern on the surface. The absorption enhancement is slightly dominant in 2D random grating pattern over 1D. This is in accordance with our previous observation of higher power diffraction into orders greater than zero by 2D random grating. Still, both gratings are having the significant effects on the absorption demonstrate the efficiency of random gratings as light trapping and super absorber structures. Same type of structures with higher refractive index and plasmonic materials may exhibit interesting and significant properties which show the wide scope of research and development possiblility with these structures.

The random grating structures are exhibiting the properties of both random medium and the diffractive elements in terms of their diffraction efficiency which are also affected by the diffusive scattering of randomness in the medium. The usage of the random textures and the diffractive elements (both dielectric and plasmonic) are crucial in reducing the reflection, increasing the path length of light inside the cell and for optical coupling^23^,^24^, for example, in the case of solar cell applications. Having both random medium and diffraction features in a single element is a clear advantage in the case of solar cells. Hence, these random grating elements may be useful for reducing reflection as surface element and at the same time spreading the light inside the cell due to its dual properties. This certainly enhances the light harvesting capability of the cell.

In conclusion, the 1D and 2D novel random gratings (Randomly distributed 1D and 2D micro grating patterns) based on individual speckle diffraction are fabricated. It is found that the 1D random grating showing the interesting and unusual diffraction pattern with optical contributions from diffraction and diffusion. The 2D random grating showing the square array diffraction pattern found to exhibit higher diffraction efficiency. As an immediate application, we have demonstrated the reduction in transmission which indicates improved absorption in thin “Si-random grating-Si” sandwich structure that can find potential solar energy harvesting applications. It is envisaged that these results lead to the fabrication of new type of structures, particularly useful in solar energy harvesting structures, detectors, light trapping (for enhanced absorption) and free form optics applications.

## Materials and Methods

The experiments are performed on the silicon substrate coated with the AZ7220 positive photoresist. The Si substrate is treated with HMDS (hexamethyldisilazane) priming for 1 minute, followed by spin coating of photoresist at 5000 rpm for 30 seconds. The thickness of the photoresist is maintained at 1.2 μm–1.5 μm. After the photo exposure the substrate is developed with MIF 826 (Metal Ion Free) developer for 1 min. In the case of higher density speckle pattern, 30 seconds is sufficient to get the good pattern followed by a thorough rinsing.

### Angular power measurement

**Figure 7:**
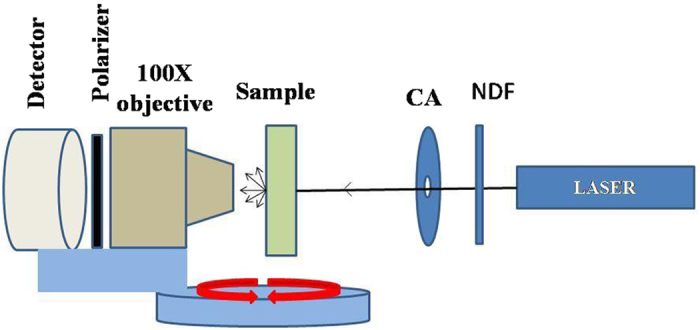
The angular power and spectral measurement setup.

The angular scattering measurements are done using the experimental set up shown in Fig. 7 on samples to find out the angular power/ spectral distribution. The laser radiation passes through the NDF (neutral density filter) and circular aperture and falls on to the sample. Here sample is a random grating where beam falls on the middle. Before the sample is fixed the alignment is done the laser beam, the objective (100×), the polarizer (Optional) and the detector are exactly kept at the same height such that the center of each optical element passes through the optic axis. The objective, polarizer and detector are mounted on circularly rotatable base and mount so that the scattered signal is collected from 90–270°. The objective window distance from sample is constant at all angles. The linearly polarized laser light/ broad band light is incident on the sample and light signal is collected at various angles by the detector^25^.

### Si-Random grating-Si sandwich structure fabrication

The amorphous Si of 1 μm thickness is coated on to the glass substrate in a well-controlled manner. The photoresist (~500 nm) is coated onto the Si material by spin coating where the thickness can be controlled by the spin speed. The random gratings are fabricated on to the photoresist by the above mentioned method followed by another coating of Si (1 μm). Here, the region where gratings are patterned led to the fabrication of Si-random grating-Si sandwich structure as illustrated.

## Additional Information

**How to cite this article**: Bingi, J. and Murukeshan, V.M. *Individual speckle diffraction* based 1D and 2D Random Grating Fabrication for detector and solar energy harvesting applications. *Sci. Rep.* **6**, 20501; doi: 10.1038/srep20501 (2016).

## Supplementary Material

Supplementary Information
