# Supplementary material for: Individual speckle diffraction based 1D and 2D Random Grating Fabrication for detector and solar energy harvesting applications
Source: Sci Rep. 2016 Feb 4;6:20501. doi: 10.1038/srep20501 (PMC4740903; doi:10.1038/srep20501)
Supplement: Supplementary Information [file srep20501-s1.pdf]

Individual speckle diffraction based 1D and 2D Random Grating Fabrication for detector and solar energy harvesting applications

Jayachandra Bingi, Murukeshan Vadakke Matham*
Center for Optical and Laser Engineering (COLE), School of Mechanical and Aerospace Engineering,
Nanyang technological University, SINGAPORE 639798
E-mail: mmurukeshan@ntu.edu.sg

Supplementary data

1. The choice of aperture shape and size:

The circular aperture with sharp edges of diameter 4 mm and 1.5 mm in the case of 1D and 2D random grating fabrication is chosen based on the following reasoning.

A circular aperture of 4mm diameter with sharp edges having thickness or the width of the edge ~600 µm is used in the set up. Our target was to produce the individual speckle diffraction patterns by reducing the number of normal speckles (un-diffracted speckles) as they can act as noise. In order to meet this requirement, a geometry where the circumference (C) comparable to the area (A) which is practically feasible in the optics configuration is chosen. When A>C, A<C, both cases found to suffer from noise (presence of a larger number of normal speckles). It is found that the circular aperture with the mentioned radius will satisfy the criteria among the different aperture shapes such as circle, square and equilateral triangle. Further, use of apertures such as square and triangle found to produce patterns affected due to their corners. This problem did not come up in the case of circular aperture as it provides same type of edge conditions at all points of the aperture to the incoming speckle.

The aperture with A<C gives the single speckle diffraction but gives small area patterns. This feature is exploited to fabricate the 2D random grating by using multiple apertures of 1.5 mm diameter in a single pupil. Here, the pupil contains 10 apertures of 1.5 mm diameter each in an area equal to 2.5 cm^2, where the diffracted speckles from each aperture form the 2D random grating patterns. Figures S1 shows the relation between area and circumference to the diameter of circular apertures (a) and to the square (b)or triangular (c) apertures, respectively.

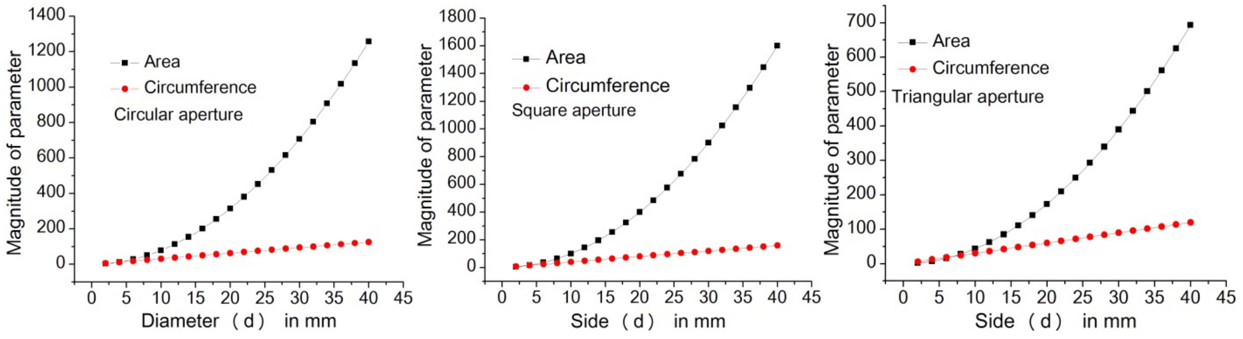

(a) (b) (c)

Figure S1: Relationship between area and circumference to the diameter of circular apertures (a) and to the square (b)or triangular (c) apertures.

2. Feature tunability of random gratings:

The size, density and spacing of the random grating are tuned by changing the mean size of the speckle and density of the speckle reaching at the lens aperture. The density of the speckles is generally controlled by varying the D-CL (Diffuser to convex lens) distance in the experimental setup. Further, the pitch size of the random grating can be tuned by varying the width of the aperture sharp edge. Figure S2 *a*, *b* show the lower and higher density random grating patterns which are recorded by choosing the D-CL distances 2.5 cm and 6 cm respectively. The pitch of the random gratings is tuned by using the aperture widths 800 µm and 600 µm to achieve the average pitch sizes of 1.08 µm and 1.45 µm, as shown in the figures S1 *c*, *d* respectively.

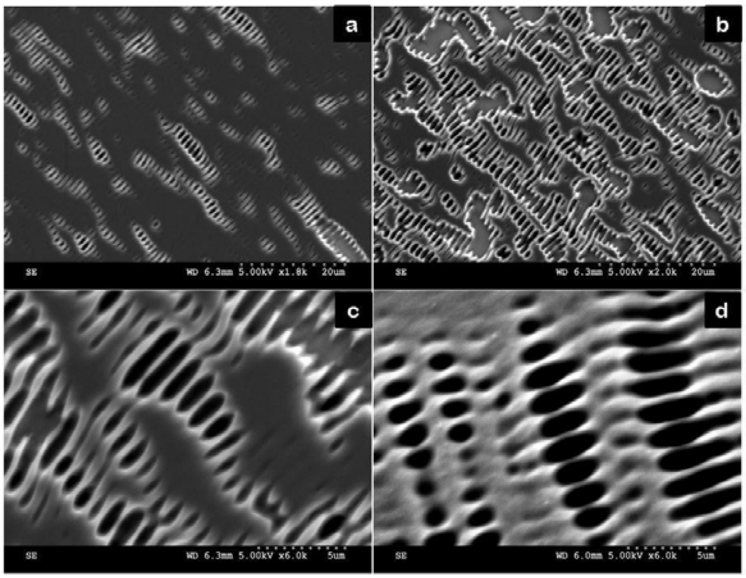

Figure S2: These SEM images show the 1D random gratings of different (a, b) density of micro gratings, (c, and d) the random grating patterns of different size.
